# A Validated Liquid Chromatographic Method for Berberine Analysis in Tissue and Application

**DOI:** 10.1155/2020/8892696

**Published:** 2020-09-30

**Authors:** Neng Zhou, Dangmei Liu, Xiaowang Bao

**Affiliations:** ^1^Guangxi Key Laboratory for Agricultural Resources Chemistry and Biotechnology, Yulin 537000, China; ^2^Colleges and Universities Key Laboratory for Efficient Use of Agricultural Resources in the Southeast of Guangxi, Yulin, China; ^3^College of Chemistry and Food Science, Yulin Normal University, Yulin, China

## Abstract

Simple and rapid high-performance liquid chromatography methods were developed for the determination of berberine (BB) in various rat tissues so as to evaluate a P-gp inhibitor, glycyrrhetinic acid (GA), on BB's oral bioavailability. Acetonitrile was used to extract BB from tissues and showed different extraction recoveries in diverse tissues. The intra- and interday precision and accuracy were less than 10%. Long-term stability, pre (post) -preparative stability, and freeze-thaw stability were evaluated, and the results showed it could meet the need of this study. The proposed methods were subsequently applied to investigate the possible drug-drug interaction of GA and BB in vivo from the aspect of tissue distribution. The results showed that no significant difference was found between the group of low dose and high dose at the same time point. The tissue distributions show a saturated model, i.e., the content of BB in tissue tends to be constant while its dose is more than 200 mg/kg. Besides, the contents of BB ranged from high to low according to the order of the liver, kidney, and spleen. The BB content in the liver is especially high as compared to other tissues.

## 1. Introduction

Berberine (BB) is an isoquinoline alkaloid, which is also the main ingredient of some traditional Chinese medicines (TCMs), such as Phellodendri Cortex, Coptidis Rhizoma, and Radix Berberidis [1, 2]. Glycyrrhizic acid (GL), a triterpenoid, is one of the characteristic ingredients of licorice which is the most commonly used TCM in prescription. Glycyrrhetinic acid (GA), namely, the metabolite of GL, is the mainly detected form in plasma during oral administration of GL or Licorice [3, 4]. Moreover, as main ingredients of plant medicine, GA and BB have been found to have various pharmacological activities. For GA, anti-inflammatory [5, 6], antivirus [7], hepatotoxin protection [8], antiulcer [9], antitumor [10, 11], and adrenal cortical hormone kind function [12] have been reported. Clinical trials have clearly shown that GA has a good effect on all types of dermatitis [13] and purulent scar disease [14]. As to BB, antibacterial [15], antitumor [16, 17], antioxidant [17, 18], anti-inflammatory [19], and cholesterol-lowering [20–22] effects have been documented. BB has been proved its potential in future clinic application for hyperlipidemia [21], diabetes [24], and cardiovascular [23] and neuroprotective diseases [25].

In prescription of TCM, licorice may combine with *Coptis chinensis* Franch [26, 27], which is now commonly utilized for the treatment of general pyrexia, diabetes mellitus, hyperlipemia, and diarrhea in clinics [28]. It had been reported that GA could react with BB (acid-base reaction) and produce a precipitate in vitro [29]. This may exert an effect on the bioavailability of BB and/or GA when both of these components exist in the same prescription. Besides, GA has been believed to play a synergistic role with other ingredients in prescription of TCMs during treatment. Moreover GA is reported to be an inhibitor of P-glycoprotein and multidrug resistance protein 1 [30] and BB is also a substrate of P-gp [31]. However, there are no statistics available to address the effects of GA on the tissue distribution of BB in vivo. Thus, the present study is focused on the effect of oral administration of GA on the tissue distribution of BB in an animal model.

There are some methods for analysis of BB in biological samples, such as capillary electrophoresis [32, 33], a flow-injection chemiluminescence system [34], near-infrared spectroscopy [35], the UPLC–MS/MS method [36, 37], and HPLC with MS/MS [38–43] or a UV detector [44, 45]. These methods focus on the fecal sample [32], plasma [33, 36–43], and herbal or herbal preparations [34, 35, 44, 45]. A few methods pay close attention to the determination of BB in tissues [46–48], in which some needed a two-step extraction [46, 47] and one used the HPLC/MS/MS system [48]. Besides, the HPLC method is a very robust and reliable approach which is the main way for the analysis of constituents in TCMs in the China standard system. Moreover, a cheap and simple method is necessary for the evaluation of drug-drug interaction for a much complicate system such as TCM from the angle of tissue distribution. In this study, high-performance liquid chromatography methods were developed for the evaluation of the effects of GA on the tissue distribution of BB.

## 2. Materials and Methods

### 2.1. Chemicals and Animals

Berberine bought form Aladdin Chemistry Co., Ltd. (HPLC purity 97%); glycyrrhetinic acid was obtained from AR, Fluka Chemical Corp., product of Spain; methanol and acetonitrile (HPLC grade) were obtained from Amethyst Chemicals (China); and sodium carboxymethyl cellulose was obtained from Aladdin Chemistry Co., Ltd. Other reagents were of analytical level. Distilled water was used.

Seventy-two Sprague Dawley (SD, SPF level) rats (180 ± 30 g), male and female each half, were purchased from the Experimental Animal Center of Guangxi Medical University (Guangxi, China). They were kept under room temperature for 2 days before study.

### 2.2. HPLC Conditions

Chromatography detection was carried out on a CTO-20A-type Shimadzu high-performance liquid chromatograph (HPLC) with an SPD-20A and LC-20AT unit, a CTO-20AC column oven, and a Shimadzu C18 column (4.6 × 250 mm), using methanol-1% acetic acid (51 : 49) as the mobile phase; the column temperature was set at 31 degrees Celsius; the detection wavelength was 350 nm; the flow rate was 1.0 mL/min; the volume of injection was 20 *μ*L; and the elution was performed in an isocratic mode.

### 2.3. Preparation of Solutions

Berberine stock solution for the HPLC method was prepared by dissolving BB in methanol to obtain 500 ng/mL. The solution was, then, diluted with methanol to achieve standard working solutions. Quality control (QC) samples: 100 *μ*L three/two level concentration of BB was transferred to a centrifuge tube, and then, the samples were prepared according to the sample preparation procedure. High (low)-dose berberine was prepared by dissolving 2.00 g (1.00 g) BB with or without 0.25 g glycyrrhetinic acid in 0.5% sodium carboxymethyl cellulose (SCC) solution in a 50 mL volumetric flask.

### 2.4. Tissue Distribution Study

Rats in half genders were randomly divided into several groups, and each had 6 rats. Animal welfare and experimental procedures were strictly in accordance with the guide for the care and use of laboratory animals by Yulin Normal University (Grant No. YTUDW20190117). BB solutions were intragastrically administrated at a dose of 400 or 200 mg/kg body weight with or without GA. Then tissue samples were collected at 1, 2, and 4 h after administration, using six rats at each time point. The blood on the surface of tissue was washed away with normal saline and, then, dried with filter paper. These samples were stored at −20 degrees until used.

### 2.5. Sample Preparation

The frozen tissue samples were naturally thawed. Then, they were cut into several large pieces with scissors, impurities such as blood clots were carefully removed, and then, they were cut into small pieces and dried with filter paper. 0.20 g of these tissues was put into a centrifuge tube. Then, the stuff was homogenized with a homogenizer (Ningbo Xinzhi Biotechnology Co., Ltd.) by addition of 0.5 mL physiological saline. 2 mL acetonitrile was added in succession and extracted for 2 min by vortex. Then, centrifugation was performed for 5 min at a speed of 12000 r/min. 2 mL of the supernatant was transferred to another centrifuge tube and, then, dried by N_2_ flow under a 50°C water bath. The dried samples were dissolved in 0.1 mL methanol by 2 min vortex. Then, 20 *μ*L of the supernatant was directly sampled in HPLC analysis after centrifuging for 5 min at a speed of 12000 r/min.

### 2.6. Method Validation

The method was validated using rat tissues from healthy rats following the guidelines of bioanalytical method validation as issued by the FDA center for Drug Evaluation and Research. It was performed from the following aspects: specificity, linearity and sensitivity, precision and accuracy, sample stability, and extraction recovery [37, 43].

### 2.7. Data Analysis

The calibration curves (triplicate for each concentration) and relative standard deviation (RSD) were obtained by WPS Office 2019 (Beijing Jinshan office software Co., Ltd). The statistical analysis of the data of two groups (six rats in each time point) was performed with independent samples *t*-tests using the Statistical Program for Social Sciences (SPSS 17.0 for Windows).

## 3. Results and Discussion

### 3.1. Selectivity of HPLC Analysis

**Figure 1:**
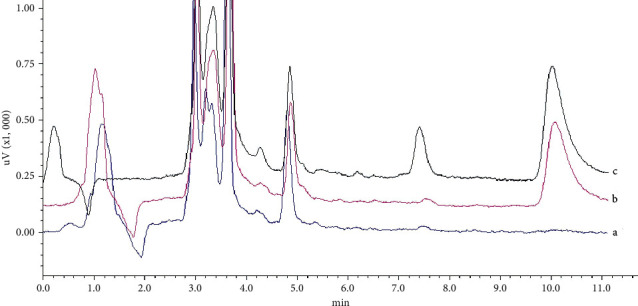
HPLC Chromatograms of BB in liver samples. Blank liver sample; (b) blank liver spiked with BB; and (c) liver sample after administration.

The selectivity of HPLC analysis was evaluated by comparing chromatograms of blank tissue samples prior to drug administration, blank tissue spiked with BB standard solution, and samples after drug administration. A good selectivity can be achieved by carefully selecting the column, mobile phase, and elution mode [36]. A reversed phase column is suitable for most cases. Under this circumstance, we tried methanol and acetonitrile and finally chose methanol-1% acetic acid (51 : 49) as the mobile phase. The typical results of the liver sample are shown in Figure 1. The run time of the HPLC method is less than 12 min, and retention time of BB is at 10.19 min. The chromatograms were free of interfering peaks from endogenetic substance at the retention time of BB. None of the interfering peaks from the endogenetic substance at the retention time of BB is found.

### 3.2. Linearity and Sensitivity

The linear relationships of the methods were evaluated by preparing seven, five, and five different concentrations of samples in the liver, kidney, and spleen using the previous extraction procedure, respectively. Three replications were made for each concentration levels. For the liver, 0.025, 1.0, 2.0, 4.0, 6.0, 8.0, and 10.0 *μ*g/g were applied. For the kidney, 0.02, 0.04, 0.10, 0.25, and 0.50 *μ*g/g were applied. For the spleen, 0.025, 0.05, 0.10, 0.25, and 0.50 *μ*g/g were used. The calibration curves were constructed by plotting the peak area of the BB against tissues concentration using the least-squares regression mode. The lower limit of detection (LOD) was the concentration giving a signal-to-noise ratio of, at least, 3-folds. The lower limit of quantitation (LOQ) was the concentration giving a signal-to-noise ratio of, at least, 10-folds. The results are shown in Table 1. Good linearity is found for the liver, spleen, and kidney sample detection. The LOQs basically meet the needs of this study. The LODs and LOQs are equivalent to that reported in the literature [49, 50].

### 3.3. Precision and Accuracy

The precision and accuracy of the method were investigated by determining QC samples at three/two different concentrations (three replicates for each concentration level) over three consecutive days. The precision of the method at each QC concentration was expressed as the relative standard deviation (RSD), and the accuracy was described as recovery (RE).The suitability of the precision and accuracy was assessed by the following criteria: the RSD should not exceed 10%, and the accuracy should be within 100 ± 10% of the actual values for QC samples. The results are presented in Table 2. As can been seen from Table 2, the precisions of the three kinds of samples in three concentration levels are lower than 3.93% and 5.87% (RSD) for within-day and between-day precision, respectively. The precision and accuracy can meet the need of this research.

### 3.4. Sample Stability

Stability of QC samples in tissues was investigated at three concentration levels under different storage conditions: long-term stability at −20°C for 2 weeks, pre (post) -preparative stability at room temperature for 4 (8) h, and three freeze-thaw cycles. The stability of samples was expressed as the relative standard deviation (RSD). The results could be found in Table 3. These results show that the samples are stable during the study. We also evaluated a 7-day stability of BB by a diluted stock solution of it. The result showed that the RSD was 1.9%.

### 3.5. Extraction Recovery

Tissue extraction usually includes single-phase extraction and biphasic extraction. The former used a water-soluble solvent, such as methanol and acetonitrile [51], while the latter applied a water-insoluble solvent, for example, ethyl acetate and dichloromethane [46, 47]. The extraction recovery of BB at three or two QC levels was conducted by calculating the ratio of the peak area of blank tissue spiked with BB and the peak area of the standard QC solution. In the liver, the extraction recovery is 74.1%, 72.2%, and 70.3% (average 72.2%) for three QC samples (*n* = 3), respectively. In the kidney, the extraction recovery is 63.7% and 59.0% (average 61.4%) for two QC samples (*n* = 3), while in the spleen, the extraction recovery is 51.3% and 48.6% (average 50.0%) for two QC samples (*n* = 3). The results show that the binding ability of these tissues to BB may be different.

### 3.6. Results of Tissues Distribution

The frozen tissue samples were naturally thawed. 0.20 g of these tissues was taken and treated in accordance with the abovementioned sample processing method. Then, 20 *μ*L volume of the prepared sample was injected for HPLC analysis doubly. The tissue distributions of BB at a dose of 400 or 200 mg/kg body weight with or without GA are shown in Tables 4 and 5. The tissue distributions are presented as microgram BB per gram tissue and calculated by calibration curves. As shown in Tables 4 and 5, no significant difference (*p* > 0.05) is found except that in the kidney 2 h after a dose of 200 mg/kg between the group with and without GA at the same time point. Moreover, no significant difference (*p* > 0.05) is found between the group of low dose and high dose at the same time point. The tissue distributions show a saturated model, i.e., the content of BB in tissue tends to be constant while its dose is more than 200 mg/kg. Besides, the contents of BB ranged from high to low according to the order of the liver, kidney, and spleen. BB in the liver is especially high as compared to other tissue.

## 4. Conclusions

During preliminary evaluation of the drug-drug in complex system such as TCM, we may judge by determining contents in the late distribution phase, equilibrium phase, and early elimination phase. In this case, where the concentration of components is relatively high, HPLC with a UV detector will achieve good results.

The validated high-performance liquid chromatography methods are simple as to sample preparation. It has good accuracy and precision and has been successfully applied to determine berberine in tissues and evaluate the effect of GA, a P-gp inhibitor, on BB's oral bioavailability. Our study reported, for the first time, the saturated distribution of BB in tissue. The tissue distribution results will provide useful information for the use of berberine in clinical trials.

## Figures and Tables

**Table 1:** The calibration curves, LOD, and LOQ of BB in different tissues (*n* = 3).

Samples	Regression equation	Correlation coefficients (*r*)	Linear range (*μ*g/g)	LOD (*μ*g/g)	LOQ (*μ*g/g)
Liver	*y* = 36033*x* − 664	0.9999	0.025–10.0	0.025	0.040
Kidney	*y* = 34212*x* − 380	0.9996	0.020–0.50	0.017	0.036
Spleen	*y* = 31092*x* − 555	0.9993	0.025–0.50	0.023	0.035

**Table 2:** Precision and accuracy of the method (*n* = 6).

Tissues	Concentration (*μ*g/g)	Within-day precision RSD (%)	Between-day precision RSD (%)	Recovery (%)
Liver	0.25	5.30	5.87	92.66
	1.25	2.37	2.53	100.1
	10.0	3.93	4.00	102.2

Kidney	0.10	0.98	1.06	107.0
	0.25	1.74	2.48	92.82

Spleen	0.10	2.82	3.63	106.7
	0.25	1.74	2.32	103.0

**Table 3:** Stability of BB in samples (*n* = 3).

Tissues	Concentration (*μ*g/g)	Freeze-thaw cycle RSD (%)	Prepreparative RSD (%)	Postpreparative RSD (%)	−20°C for 2 weeks RSD %
Liver	0.25	4.2	7.5	3.9	9.2
	1.25	3.8	6.4	4.6	9.5
	10.0	3.5	7.1	4.2	9.1

Kidney	0.10	2.1	2.7	1.3	1.1
	0.25	2.4	4.3	2.9	1.9

Spleen	0.10	3.8	4.2	3.3	3.7
	0.25	2.1	2.1	1.6	4.4

**Table 4:** Results of BB at a dose of 400 mg/kg in tissues of rat (mean ± SD, *n* = 6, *µ*g/g).

Tissue	1 h		2 h		4 h	
	With GA	Without GA	With GA	Without GA	With GA	Without GA
Liver	0.666 ± 0.142	0.629 ± 0.267	3.21 ± 1.92	2.08 ± 1.34	3.76 ± 2.73	2.42 ± 2.28
Spleen	0.049 ± 0.012	0.062 ± 0.019	0.183 ± 0.123	0.137 ± 0.109	0.349 ± 0.115	0.291 ± 0.110
Kidney	0.195 ± 0.134	0.189 ± 0.143	0.244 ± 0.120	0.267 ± 0.349	0.297 ± 0.155	0.202 ± 0.064

**Table 5:** Results of BB at a dose of 200 mg/kg in tissues of rat (mean ± SD, *n* = 6, *µ*g/g).

Tissue	1 h		2 h		4 h	
	With GA	Without GA	With GA	Without GA	With GA	Without GA
Liver	0.450 ± 0.217	0.772 ± 0.343	1.814 ± 2.003	3.734 ± 1.875	0.850 ± 0.268	0.882 ± 0.132
Spleen	0.068 ± 0.037	0.0504 ± 0.023	0.114 ± 0.171	0.324 ± 0.292	0.065 ± 0.060	0.075 ± 0.065
Kidney	0.137 ± 0.148	0.397 ± 0.315	0.193 ± 0.089*∗*	0.287 ± 0.122*∗*	0.188 ± 0.052	0.241 ± 0.259

^*∗*^ *p* < 0.05.

## Data Availability

The data used to support the findings of this study are included within the article.

## Abbreviations

BB: Berberine
GA: Glycyrrhetinic acid
HPLC: High-performance liquid chromatography
LOD: The lower limit of detection
QC: Quality control
TCM: Traditional Chinese medicine.

## References

[1] Ye M., Fu S., Pi R., He F. (2009). Neuropharmacological and pharmacokinetic properties of berberine: a review of recent research. *Journal of Pharmacy and Pharmacology*.

[2] Tsai P.-L., Tsai T.-H. (2002). Simultaneous determination of berberine in rat blood, liver and bile using microdialysis coupled to high-performance liquid chromatography. *Journal of Chromatography A*.

[3] Wang Q., Shi R., Ma Y.-M. (2013). Content determination of the major constituents of Yinchenzhufu decoction via ultra high-performance liquid chromatography coupled with electrospray ionisation tandem mass spectrometry. *Journal of Pharmaceutical and Biomedical Analysis*.

[4] Wang Y., Xu C., Wang P. (2013). Pharmacokinetic comparisons of different combinations of Shaoyao-Gancao-Decoction in rats: simultaneous determination of ten active constituents by HPLC-MS/MS. *Journal of Chromatography B*.

[5] Amagaya S., Sugishita E., Ogihara Y., Ogawa S., Okada K., Aizawa T. (1984). Comparative studies of the stereoisomers of glycyrrhetinic acid on anti-inflammatory activities. *Journal of Pharmacobio-Dynamics*.

[6] Gao Z., Kang X., Xu C. (2011). Research progress of anticancer mechanism of glycyrrhetinic acid. *China Journal of Chinese Materia Medica*.

[7] Wang J., Sun Q., Gao P. (2011). Bioconversion of glycyrrhizinic acid in liquorice into 18-betaglycyrrhetinic acid by aspergillus parasiticus speare BGB. *Applied Biochemistry and Microbiology*.

[8] Kiso Y., Tohkin M., Hikino H., Hattori M., Sakamoto T., Namba T. (1984). Mechanism of antihepatotoxic activity of glycyrrhizin, I: effect on free radical generation and lipid peroxidation. *Planta Medica*.

[9] Yano S., Harada M., Watanabe K. (1989). Antiulcer activities of glycyrrhetinic acid derivatives in experimental gastric lesion models. *Chemical & Pharmaceutical Bulletin*.

[10] Huang R. Y., Chu Y. L., Huang Q. C. (2014). 18*β*-glycyrrhetinic acid suppresses cell proliferation through inhibiting thromboxane synthase in non-small cell lung cancer. *PLoS One*.

[11] Huang Y.-C., Kuo C.-L., Lu K.-W. (2016). 18*α*-Glycyrrhetinic acid induces apoptosis of HL-60 human leukemia cells through caspases- and mitochondria-dependent signaling pathways. *Molecules*.

[12] Mazzocchi G., Rossi G. P., Neri G., Malendowicz L. K., Albertin G., Nussdorfer G. G. (1998). 11*β*-Hydroxysteroid dehydrogenase expression and activity in the human adrenal cortex. *The FASEB Journal*.

[13] Saeedi M., Morteza-Semnani K., Ghoreishi M. R. (2003). The treatment of atopic dermatitis with licorice gel. *Journal of Dermatological Treatment*.

[14] Li X.-L., Zhou A.-G., Zhang L., Chen W.-J. (2011). Antioxidant status and immune activity of glycyrrhizin in allergic rhinitis mice. *International Journal of Molecular Sciences*.

[15] Subbaiah T. V., Amin A. H. (1967). Effect of berberine sulphate on Entamoeba histolytica. *Nature*.

[16] Ho Y.-T., Yang J.-S., Lu C.-C. (2009). Berberine inhibits human tongue squamous carcinoma cancer tumor growth in a murine xenograft model. *Phytomedicine*.

[17] Abd El-Wahab A. E., Ghareeb D. A., Sarhan E. E. (2013). In vitro biological assessment of berberis vulgaris and its active constituent, berberine: antioxidants, anti-acetylcholinesterase, anti-diabetic and anticancer effects. *BMC Complementary and Alternative Medicine*.

[18] Hwang J.-M., Wang C.-J., Chou F.-P. (2002). Inhibitory effect of berberine on tert -butyl hydroperoxide-induced oxidative damage in rat liver. *Archives of Toxicology*.

[19] Kuo C.-L., Chi C.-W., Liu T.-Y. (2004). The anti-inflammatory potential of berberine in vitro and in vivo. *Cancer Letters*.

[20] Zhang H., Wei J., Xue R. (2010). Berberine lowers blood glucose in type 2 diabetes mellitus patients through increasing insulin receptor expression. *Metabolism*.

[21] Kong W., Wei J., Abidi P. (2004). Berberine is a novel cholesterol-lowering drug working through a unique mechanism distinct from statins. *Nature Medicine*.

[22] Zhang Y., Li X., Zou D. (2008). Treatment of type 2 diabetes and dyslipidemia with the natural plant alkaloid berberine. *The Journal of Clinical Endocrinology & Metabolism*.

[23] Ko B.-S., Choi S. B., Park S. K., Jang J. S., Kim Y. E., Park S. (2005). Insulin sensitizing and insulinotropic action of berberine from cortidis rhizoma. *Biological & Pharmaceutical Bulletin*.

[24] Wang Y. X., Yao X. J., Tan Y. H. (1994). Effects of berberine on delayed after depolarizations in ventricular muscles in vitro and in vivo. *Journal of Cardiovascular Pharmacology*.

[25] Stein T. D., Anders N. J., De Carli C. (2004). Neutralization of transthyretin reverses the neuroprotective effects of secreted amyloid precursor protein (APP) in APPSW mice resulting in tau phosphorylation and loss of hippocampal neurons: support for the amyloid hypothesis. *Journal of Neuroscience*.

[26] Dong Y. L., Li X. L., Chen J. P. (2017). Research on intestinal permeability of glycyrrhetinic acid in multicomponent environment. *Chinese Traditional and Herbal Drugs*.

[27] An R., Zhang H., Zhang Y. Z. (2012). Intestinal absorption of different combinations of active compounds from Gegenqinlian decoction by rat single pass intestinal perfusion in situ. *Acta Pharmacologica Sinica*.

[28] Liu T., Cui Y., Tian X. (2017). Detection of chemical constituents in Gegenqinlian decoction by ultra-high performance liquid chromatography coupled with Fourier transform ion cyclotron resonance mass spectrometry. *Analytical Methods*.

[29] Wu Y. Y., Yuan H. Y., Chen X. H. (2014). Content change of main components of coptis rhizome and scutellaria Radix couples among different compatibilities of medicinal materials in Xiexin decoction and gegen Qinlian decoction. *Chinese Journal of Experimental Traditional Medical Formulae*.

[30] Nabekura T., Yamaki T., Ueno K., Kitagawa S. (2008). Inhibition of P-glycoprotein and multidrug resistance protein 1 by dietary phytochemicals. *Cancer Chemotherapy and Pharmacology*.

[31] Qiu W., Jiang X. H., Liu C. X., Ju Y., Jin J. X. (2009). Effect of berberine on the pharmacokinetics of substrates of CYP3A and P-gp. *Phytotherapy Research*.

[32] Chu C., Lian L. M., Liu C. J. (2020). Online preconcentration by electrokinetic supercharging for sensitive determination of berberine and jatrorrhizine in biological samples. *Biomedical Chromatography*.

[33] Song T. H., Zhang K. Y., Lao L. X. (2014). Simultaneous determination of berberine and palmatine in human plasma and in urine by capillary electrophoresis combined with polypropylene hollow fiber liquid–liquid–liquid microextraction. *Analytical Methods*.

[34] Liang Y.-D., Yu C.-X. (2013). Determination of berberine in pharmaceutical preparations using acidic hydrogen peroxide-nitrite chemiluminescence system. *Drug Testing and Analysis*.

[35] Zhang S. Y., Chen M., Chen Y. (2020). Rapid and simultaneous determination of moisture and berberine content in coptidis rhizoma and phellodendri chinensis cortex by near-infrared spectroscopy and chemometrics. *Journal of Innovative Optical Health Sciences*.

[36] Li G., Yang F., Liu M., Su X., Zhao M., Zhao L. (2016). Development and application of a UPLC-MS/MS method for simultaneous determination of fenofibric acid and berberine in rat plasma: application to the drug-drug pharmacokinetic interaction study of fenofibrate combined with berberine after oral administration. *Biomedical Chromatography*.

[37] Liu M., Su X., Li G., Zhao G., Zhao L. (2015). Validated UPLC-MS/MS method for simultaneous determination of simvastatin, simvastatin hydroxy acid and berberine in rat plasma: application to the drug-drug pharmacokinetic interaction study of simvastatin combined with berberine after oral administration in rats. *Journal of Chromatography B*.

[38] Liu F., Li Z., Shi X., Zhong M. (2011). Determination of berberine, palmatine and jatrorrhizine in rabbit plasma by liquid chromatography-electrospray ionization-mass spectrometry. *Journal of Pharmaceutical and Biomedical Analysis*.

[39] Gupta P. K., Hubbard M., Gurley B., Hendrickson H. P. (2009). Validation of a liquid chromatography-tandem mass spectrometric assay for the quantitative determination of hydrastine and berberine in human serum. *Journal of Pharmaceutical and Biomedical Analysis*.

[40] Feng J., Xu W., Tao X. (2010). Simultaneous determination of baicalin, baicalein, wogonin, berberine, palmatine and jatrorrhizine in rat plasma by liquid chromatography-tandem mass spectrometry and application in pharmacokinetic studies after oral administration of traditional Chinese medicinal preparations containing scutellaria-coptis herb couple. *Journal of Pharmaceutical and Biomedical Analysis*.

[41] Fei Y., Rui Y. (2011). Simultaneous determination of berberine and evodiamine in dog plasma by LC-ESI-MS method and its application to pharmacokinetics. *Journal of Chromatographic Science*.

[42] Deng Y., Liao Q., Li S., Bi K., Pan B., Xie Z. (2008). Simultaneous determination of berberine, palmatine and jatrorrhizine by liquid chromatography-tandem mass spectrometry in rat plasma and its application in a pharmacokinetic study after oral administration of coptis-evodia herb couple. *Journal of Chromatography B*.

[43] Liu G., He W., Cai H. (2014). The simultaneous determination of berberine, palmatine, coptisine, epiberberine and jatrorrhizine in rat plasma by LC-MS/MS and a pharmacokinetic comparison after the oral administration of Rhizoma coptidis and Jiao-Tai-Wan extract. *Analytical Methods*.

[44] Li L., Long W., Wan X., Ding Q., Zhang F., Wan D. (2015). Studies on quantitative determination of total alkaloids and berberine in five origins of crude medicine “sankezhen”. *Journal of Chromatographic Science*.

[45] Ma L., Li J.-q., Hu Y.-d. (2015). Determination of berberine in phellodendron amurense from different sites of Changbai mountain. *Journal of Forestry Research*.

[46] Yan Q. N., Zhang S., Zhang Z. Q. (2009). Study on the tissue distribution of berberine from rhizoma coptidis and compatibility with rhizoma coptidis and cortex cinnamomi in rats. *Journal Of Chinese Medicinal Materials*.

[47] Hu Y. L., Chen C., Zou Z. Y. (2014). Comparative study of pharmacokinetics and tissue distribution of 8-cetylberberine and berberine in rats. *Acta Pharmaceutica Sinica*.

[48] Lu M., Chu Z., Wang L. (2020). Pharmacokinetics and tissue distribution of four major bioactive components in rats after oral administration of Xianglian pill. *Biomedical Chromatography*.

[49] Li B. X., Yang B. F., Hao X. M. (2000). Study on the pharmacokinetics of berberine in single dosage and coadministration with oryzanol in rabbits and healthy volunteers. *Chinese Pharmaceutical Journal*.

[50] Zhou N., Zou C. Y., Qin M. L. (2019). A simple method for evaluation pharmacokinetics of glycyrrhetinic acid and potential drug-drug interaction between herbal ingredients. *Scientific Reports*.

[51] Dewangan G., Mishra A., Mandal T. K. (2016). A simple RP-HPLC method for determining imidacloprid residues in goat tissues. *Biomedical Research*.

